# Environmental hygiene and healthcare-associated infection: a time-series study based on generalized additive model

**DOI:** 10.3389/fpubh.2025.1592700

**Published:** 2025-10-13

**Authors:** Renhua Li, Zhongjie Wang, Mingqi Huang, Daiying Liao, Zhe Yuan, Keli Qian

**Affiliations:** Department of Infection Control, The First Affiliated Hospital of Chongqing Medical University, Chongqing, China

**Keywords:** healthcare-associated infection, environmental hygiene, time-series analysis, generalized additive model, lag effect

## Abstract

**Objective:**

To quantitatively analyze the impact of environmental hygiene on the occurrence of healthcare-associated infection (HAI).

**Methods:**

Monitoring data of HAI and environmental hygiene from a tertiary first-class hospital from July 1, 2022, to December 31, 2024, were collected, and the impact of environmental bacterial colony forming unit (CFU) on the occurrence of HAI was analyzed by a time-series generalized additive model (GAM).

**Results:**

The single-contamination model showed a significant positive correlation between HAI and total colony count, high-touch surface (HTS) colony count, and staff' hands colony count. The same pattern was observed in the multi-contamination model.

**Conclusion:**

There is a significant correlation between environmental hygiene and the occurrence of HAI.

## Introduction

Healthcare-associated infections (HAIs) represent a persistent global public health challenge in medical institutions over the past decade, contributing significantly to elevated morbidity, mortality rates, and healthcare system expenditure (1–3). World Health Organization (WHO) surveillance data indicate that 7%−15% of hospitalized patients acquire at least one HAI during their hospital stay, with fatal outcomes occurring in 10% of affected cases (4). Comprehensive research has identified multiple hospital environmental factors as potential reservoirs for pathogenic microorganisms, including staff hand surfaces, high-touch fomites, medical instrumentation, and water distribution systems (5–9). Notably, multidrug-resistant organisms (MDROs) such as methicillin-resistant *Staphylococcus aureus* (MRSA), vancomycin-resistant Enterococci, and carbapenem-resistant Gram-negative bacteria demonstrate frequent environmental persistence in healthcare settings (10). Empirical evidence reveals these pathogens can maintain viability for extended periods exceeding decades in medical environments, with demonstrated capacity to form desiccated biofilm matrices. Such biofilm formation and subsequent microbial proliferation have been epidemiologically linked to HAI outbreak events (11).

Current infection control protocols, as outlined in international guidelines and domestic regulations, emphasize environmental sanitation as a critical intervention for HAI mitigation (12). This comprehensive approach encompasses standardized protocols for cleaning tool selection, validated disinfection methodologies, and optimized decontamination frequency (13). In Chinese healthcare institutions, environmental sanitation services predominantly operate under third-party contractual agreements. However, implementation efficacy remains suboptimal due to systemic challenges including variable workforce education levels among cleaning personnel and deficiencies in quality assurance mechanisms.

The existing evidence base establishing environmental contamination-HAI correlations primarily derives from microbial surveillance conducted during nosocomial infection outbreaks. A notable research gap persists regarding quantitative associations between routine environmental bioburden and baseline HAI incidence in non-outbreak scenarios (14, 15). Furthermore, while multiple determinants influence environmental hygiene outcomes—including cleaning material selection and staff training protocols—current studies predominantly analyze these variables as composite bundles rather than isolating individual factors (16). This methodological limitation constrains precise evaluation of specific intervention impacts on infection rates.

This investigation was conducted at a tertiary public teaching hospital (4,616-bed capacity) in Western China, utilizing two inpatient branches (A, B) as study sites. All branches maintained strict adherence to standardized infection control protocols issued by the National Health Commission of China, including the “Regulations for Cleaning and Disinfection Management of Environmental Surface in Healthcare” (17) and institution-specific “Standard Operating Procedure for Environmental Cleaning.” Identical staff training programs were implemented across all study units.

This investigation is structured to address three principal research objectives through systematic methodological implementation: (1) comparative evaluation of disinfection efficacy between distinct reusable cleaning equipment decontamination protocols; (2) quantitative characterization of environmental microbiome bioburden fluctuations associated with differing intervention strategies; and (3) establishment of epidemiological correlations between measurable microbial contamination parameters and healthcare-associated infection incidence patterns. The study framework enables rigorous examination of intervention effectiveness, microbial dynamics, and clinical outcomes through integrated analytical approaches.

The experimental framework provides novel insights into optimization of environmental hygiene protocols through evidence-based disinfection strategy selection, with particular relevance for healthcare systems utilizing outsourced sanitation services.

## Methods

### Study design

A comparative observational study was conducted across two branches (Branch A and B) of a tertiary hospital between July 1, 2022 and December 31, 2024. Branch A comprises 3,200 inpatient beds, while Branch B operates 611 beds. The study population included all 477,831 hospitalized patients admitted to both branches during the observation period, alongside 9,167 environmental health samples collected from staff hands, air, and high-frequency contact surfaces. Additionally, reusable cleaning tools subjected to distinct disinfection protocols were analyzed as study objects: Branch A employed manual cleaning with chemical disinfection, while Branch B utilized centralized thermal disinfection systems (automated washer-disinfector technology). This controlled variation enables isolation of cleaning tool decontamination methodology as the primary experimental variable for comparative analysis of environmental microbial load and subsequent HAI incidence.

### Bacterial detection in reusable cleaning tools

A total of 100 post-disinfection samples of reusable cleaning textiles (including hand towels and floor towels) were randomly selected from both branches for microbiological analysis. Specimens were processed using standardized aseptic techniques: each sample was sectioned into 1 cm × 3 cm segments using sterile instruments and immersed in 5 ml of sterile normal saline containing appropriate neutralizers. After vortex mixing, 1 ml of the eluate was transferred to a sterile Petri dish, combined with 15–18 ml of nutrient agar (45–48 °C), and homogenized. Plates were incubated at 37 °C for 48 h. Finally, calculate the colony-forming units contained in each square centimeter (CFU/cm^2^) of reusable cleaning textiles samples.

### Data collection

Hospital-acquired infection (HAI) diagnoses adhered to the Diagnostic Criteria for Nosocomial Infection (Trial Edition). Monthly HAI incidence rates and case-specific data (gender, age, hospitalization duration, ward assignment) were retrospectively extracted from the Nosocomial Infection Surveillance Database (NISD). Concurrently, environmental hygiene metrics, encompassing staff hand hygiene, air quality, and high-touch surface (HTS) were obtained from the Environmental Hygiene Monitoring System. Monitoring covered all clinical wards and critical units surgical center, hemodialysis center, endoscopy suite, pharmacy intravenous admixture service, and central sterile supply department). Data collection was executed by certified infection prevention and control (IPC) personnel under strict confidentiality protocols, with subsequent quality verification and archival management by designated personnel.

### Statistical analysis

Descriptive statistics (mean, standard deviation, median, range, and interquartile range) characterized central tendencies and dispersion of monthly microbial colony-forming units per dish (MCFU/Dish) and HAI frequencies. Normality of HAI case distribution was assessed via the Kolmogorov–Smirnov test. Spearman's rank correlation analyzed associations between MCFU/Dish and HAI incidence. A generalized additive model (GAM) evaluated the non-linear relationship between environmental microbial loads and HAI occurrence. All analyses were performed using R software (v4.3.1), with statistical significance defined as *p* ≤ 0.05 (two-tailed).

## Results

### Microbial contamination of reusable cleaning tools

**Figure 1:**
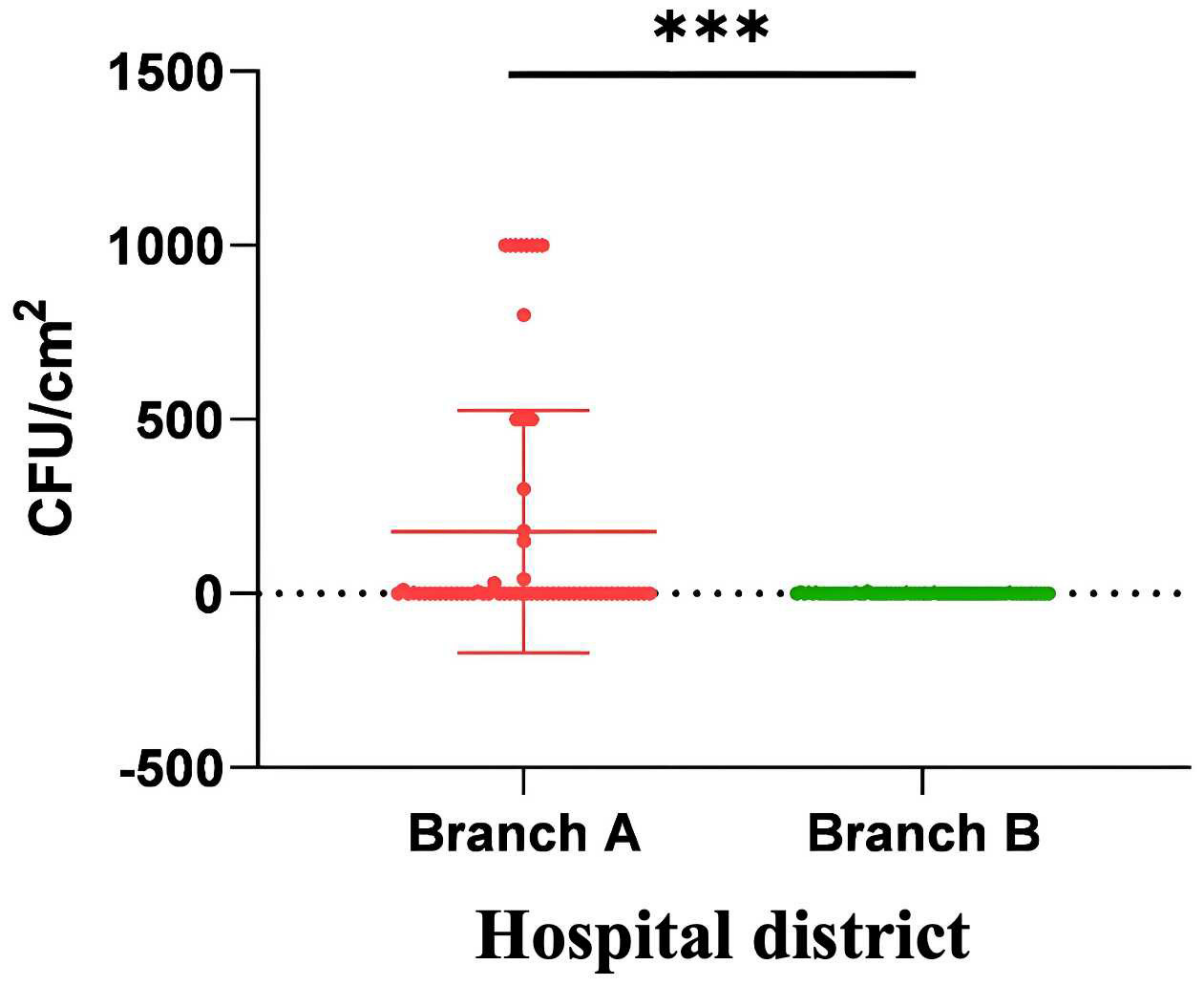
The microbiological monitoring comparison of reusable cleaning tools for fabrics in two branches. The bacterial colony counts on reusable sanitary ware cleaned manually were significantly higher than those on reusable sanitary ware cleaned by centralized thermal cleaning. ^***^p < 0.001.

Reusable cleaning textiles subjected to centralized thermal disinfection demonstrated a statistically significant reduction in bacterial colonization compared to manually processed counterparts (0.53 vs. 177.3 CFU/cm^2^; *p* < 0.001; Figure 1).

### Epidemiological profile of hospital-acquired infections

Among 477,831 hospitalized patients across both branches during the 30-month observation period, 7,455 HAIs were documented, yielding a cumulative incidence rate of 1.56% (mean: 248.5 cases/month). Branch A exhibited a significantly higher HAI incidence than Branch B (1.59 vs. 1.26%; *p* < 0.001; Table 1).

**Table 1:** Comparison of hospital infection incidence rates between the two branches.

**Hospital district**	**HAI**	**Total inpatients**	**The rate of HAI**	***p*-value**
Branch A	6,859	430,385	1.59%	< 0.001
Branch B	596	47,446	1.26%	

### Environmental hygiene monitoring outcomes

Of 9,167 environmental samples collected, 9,039 met inclusion criteria after excluding 128 contaminated or control-positive specimens. Sampling distribution included 2,308 hand hygiene evaluations (1,806 hygienic hand disinfection, 502 surgical hand disinfection), 4,138 air quality assessments, and 2,593 HTS analyses. The results revealed that except for the HTS colony count in the Branch A which was higher than that in the Branch B, there was no difference in the other two indicators (Table 2).

**Table 2:** Comparison of monitoring data on environmental sanitation between the two branches.

**Hospital district**	**Total colony count**	**HTS colony count**	**Staff' hands colony count**	**Air colony count**
Branch A	0.31 (0.03, 0.15)	0.23 (0.12, 0.47)	0.06 (0, 0.99)	0.04 (0, 0.13)
Branch B	0.22 (0.07, 0.50)	0.09 (0.03, 0.15)	0.06 (0, 0.52)	0.02 (0, 0.19)
*p-*value	0.071	0.001	0.056	0.551

### Association between HAI incidence and environmental contamination

**Figure 2:**
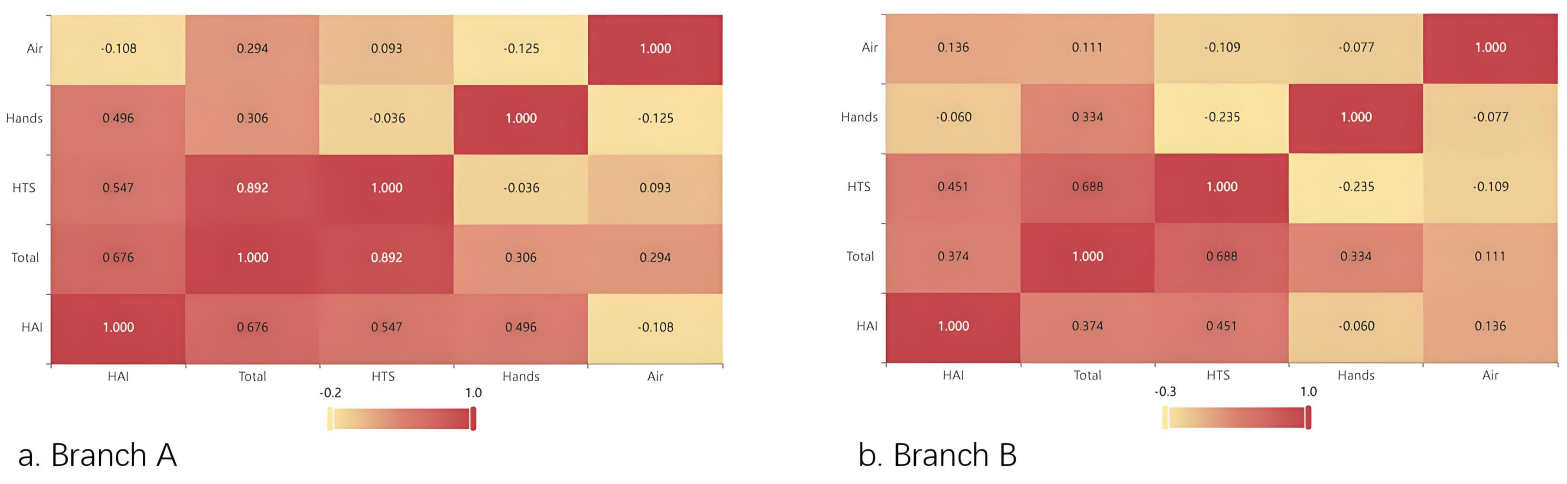
Heatmap of the correlation between environmental monitoring indicators and the monthly occurrence numbers of HAI cases. **(a)** Branch A. **(b)** Branch B.

After applying the Kolmogorov–Smirnov test, the monthly hospital infection occurrence numbers in both hospital branches were found to conform to the Poisson distribution (Za = 0.132, Pa = 0.191, Zb = 0.164, Pb = 0.058). Spearman correlation analysis revealed that the total environmental colony count (*r* = 0.676), HTS colony count (*r* = 0.547), and the colony count on the hands of staff (*r* = 496) in Branch A were positively correlated with the monthly hospital infection count (*p* < 0.05). While in Branch B, the total environmental colony count (*r* = 0.374) and HTS colony count (*r* = 0.451) were positively correlated with the monthly number of hospital infection cases (*p* < 0.05); the air colony counts in both branches were not correlated with the monthly hospital infection count (*p* > 0.05; Figure 2).

### GAM model

After adjusting for long-term trends and seasonal variability, GAM analysis identified significant associations between microbial loads and HAI incidence. The β1 of the Total colony counts, HTS colony counts, staff's hands colony counts and air colony count were shown in Table 3. We found that the total colony count, HTS colony count, and staff' hands colony count all had an impact on the monthly number of hospital infections in both hospital branches. The single-factor model indicated that the total colony count, HTS colony count, and staff hand colony count had a significant positive impact on the monthly number of hospital infections. For example, in Hospital Branch A, the β1 for the total colony count was 0.21 (*p* < 0.001), and it was estimated that the risk of hospital infection would increase by approximately 23% (95% CI) for each increase of 1 IQR.

**Table 3:** The GAM model coefficients of environmental monitoring indicators for the two hospital branches.

**Item**	β**1**		***p-*** **value**		**IRR**	
	**Branch A**	**Branch B**	**Branch A**	**Branch B**	**Branch A**	**Branch B**
Total colony count	0.21	0.15	< 0.001	0.032	1.23	1.18
HTS colony count	0.15	0.21	0.012	0.008	1.19	1.12
Staff' hands colony count	0.18	0.18	0.003	0.018	1.28	1.24
Air colony count	0.05	0.12	0.153	0.45	–	–

The lag effect of 0–6 months shows that the lag time of total colony count is the longest (3 months in Branch A and 2 months in Branch B), the lag time of HTS colony count is 1 month in both areas, while there is no lag effect for the colony count on the hands of staff in both areas. This suggests that in the influence of the environment on hospital infections, the contamination level of staff hands has an immediate effect on the occurrence of hospital infections, and the implementation of hand hygiene for staff should be strengthened. However, the total environment, especially the HTS colony count, has a lag effect on the occurrence of hospital infections, indicating that the cleaning and disinfection of environmental surfaces should be strengthened, especially the terminal disinfection of the environment, to reduce the possibility of subsequent hospital infections in inpatients.

Based on the single-pollution model, we further established a multi-pollution model by adding a tensor product smoother to capture potential non-linear interaction relationships regarding environmental pollution factors and hospital infections. The interaction effect among the total environmental colony count, HTS colony count, and the colony count on staff hands showed that the interaction term te had a statistically significant impact on the monthly occurrence of hospital infections (edf = 7.366, *p* = 0.0313).

## Discussion

In this study, the bacterial monitoring load of reusable sanitary ware using different cleaning and disinfection methods was compared, and the environmental hygiene and nosocomial infection monitoring data of the hospital for 30 month were comprehensively analyzed and evaluated.

Unsurprisingly, it was found that the bacterial load of manually cleaned reusable sanitary ware was significantly higher than that of centralized thermal cleaning, and the difference was statistically significant. The reasons for the poor effect of manual cleaning reusable sanitary ware mainly include the following two points: first, it is mainly operated by cleaning personnel, and there are disadvantages such as large individual behavior, poor implementation of norms and limited operating space; second, due to the large number of cleaning personnel involved and the wide distribution area, there are difficulties in supervision. Correspondingly, the centralized thermal cleaning and disinfection is mainly operated by the machine, the procedure is standardized, and the personnel involved and the area are centralized, which is more convenient for supervision and management. Reusable sanitary ware is the main tool for medical institutions to maintain environmental hygiene (18). Manually cleaned reusable sanitary ware may be contaminated before use, may lead to incomplete environmental cleaning, eventually excessive bacterial load on the surface of the environment. In this study, it was found that the unqualified rate of high frequency contact surface bacteria monitoring in the branch with manual cleaning was significantly higher than that in the branch with centralized heat cleaning, which also confirmed this. We also found that there were differences in the average HTS colony counts between the two hospital branches. The average HTS colony count of the district adopting centralized hot water cleaning was lower than that of the district using manual cleaning. This further indicates the direct impact of the cleaning methods for reusable sanitary fixtures on the hospital environment.

In this study, we found that the total colony count, HTS colony count and the Staff' hands colony count were positively correlated with the monthly number of hospital-acquired infections. Lee et al. (19) have shown that increased hand sanitizer use is associated with decreased rates of HAI and healthcare-associated methicillin-resistant *Staphylococcus aureus* (HA-MRSA) infections. However, a recent study shows that ICU nurses must spend 17% of their working time performing proper hand hygiene, and a 100% hand hygiene compliance rate seems unattainable (20). Numerous studies have proposed that high-frequency contact surfaces of objects are directly related to the occurrence of hospital-acquired infections, especially in circumstances such as the COVID-19 pandemic and outbreaks of multi-drug resistant bacteria (21, 22). Russotto et al.'s (23) research demonstrated that if medical staff fail to correctly perform hand hygiene before and after contacting patients, it might increase the risk of environmental contamination and subsequent healthcare-associated infections (HAIs) for patients (23). Healthcare workers can cause contamination not only through direct contact with patients but also by touching inanimate surfaces and equipment in the patients' surroundings, and bacteria can survive on dry surfaces for several month. A study that screened the hands of healthcare workers and high-frequency contact areas found that the isolated yeasts underwent cross-hospital transmission, and the same clone strain was detected in the blood specimens of patients with candidemia (24). Hence, for infection control personnel, it is particularly crucial to effectively enhance the compliance and accuracy of hand hygiene among medical staff.

The average number of colonies on high-frequency contact surfaces in our hospital is lower than the requirements stipulated in the national standard GB15982 of China (25). This is attributed to the standard operating procedures and strict implementation of environmental cleaning and disinfection regulations we have adopted. However, it was still found that it has an impact on the monthly occurrence rate of nosocomial infections. This is consistent with the findings of Schmidt et al. Even when the cleaning team strictly implements cleaning measures, the microbial load on the patient's bed surface may still exceed the standard concentration, and the microbial load increases with the increase of the patient's hospitalization time (26). This may be related to the persistent existence of biofilms on dry surfaces in medical institutions (27). Maillard proposed that the use of specialized microbial cleaners can stabilize the contamination degree of object surfaces (28). Therefore, the cleaning of high-frequency contact surfaces needs to be actively and continuously implemented, and the monitoring of the colony count should be strengthened to provide comprehensive monitoring data for medical institutions and to formulate targeted intervention measures accordingly.

This study utilized a generalized additive model (GAM) to analyze the association between environmental contamination and HAI incidence, which offers flexibility in capturing non-linear relationships and adjusting for underlying temporal trends such as seasonality and long-term changes. By incorporating a smoothing function of time, the model effectively controlled for these confounding patterns, thereby providing a more accurate estimation of the independent effects of environmental microbial loads on HAI risk. This approach is particularly suited for analyzing complex time-series data in hospital epidemiology, where infection rates may fluctuate due to both environmental factors and unobserved temporal influences. The results of both the single-factor model and the multiple factors model showed that the smoothing term s (month) of the time series was statistically significant (*p* < 0.001), indicating that there was a significant non-linear relationship between the occurrence of HAI and the month, which consistent with the research result of Roux. While the non-linear relationship may be more influenced by ambient temperature and humidity, air quality or other seasonal factors (29). Recently, there has been an application of the GAM model in the research related to HAI (30), which proves that GAM has a promising application in the field of HAI prevention and control.

Furthermore, the lag effect analysis revealed distinct temporal patterns between different contamination sources. The colony count on staff hands exhibited an immediate effect on HAI occurrence, indicating that hand hygiene interventions should be implemented in real time to minimize transmission risks. In contrast, the total environmental colony count and HTS colony count demonstrated lag effects of up to 3 months and 1 month, respectively, suggesting that environmental surface contamination accumulates over time and influences HAI incidence in a delayed manner. This finding underscores the importance of reinforcing terminal disinfection protocols and scheduled environmental cleaning to mitigate residual microbial burden. Consequently, infection control strategies should prioritize immediate hand hygiene compliance alongside structured environmental decontamination schedules to address both instantaneous and delayed contamination pathways effectively.

This study has some limitation. First, as the study is a single-center research, its results have certain limitations in terms of their generalizability. Second, it is a retrospective analysis involving data over a period of 30 months, there may be potential bias in the test results due to the reasons related to specimen collection. In the future, it is urgently necessary to optimize and improve the model and methods of this study through multi-center and prospective research.

## Conclusions

To sum up, the innovation of this study lies in the fact that it is the first time to apply GAM to explore the association between environmental pollution factors and the risk of HAI. The GAM model has been extended to the interdisciplinary research field of environmental hygiene and hospital infection prevention and control. Moreover, the types of environmental specimens included in this study cover the types usually monitored in medical institutions. Using 30-month data for in-depth modeling analysis, it provides a new perspective for the formulation and implementation of HAI prevention and control strategies.

## Data Availability

The original contributions presented in the study are included in the article/supplementary material, further inquiries can be directed to the corresponding author.
